# The Social Construction of Aging Among a Clinic-Based Population and Their Healthcare Workers in Zambia

**DOI:** 10.3389/ijph.2024.1606607

**Published:** 2024-04-22

**Authors:** Anjali Sharma, Chanda Mwamba, Natalie St Clair-Sullivan, Belinda V. Chihota, Jake M. Pry, Carolyn Bolton-Moore, Michael J. Vinikoor, Guy K. Muula, Harriet Daultrey, Joel Gittelsohn, Lloyd. B. Mulenga, Namasiku Siyumbwa, Gilles Wandeler, Jaime H. Vera

**Affiliations:** ^1^ Centre for Infectious Disease Research in Zambia, Lusaka, Zambia; ^2^ Brighton and Sussex Medical School, Brighton, United Kingdom; ^3^ Department of Preclinical Medicine, Faculty of Medicine, Institute for Social and Preventive Medicine, University of Bern, Bern, Switzerland; ^4^ School of Medicine, University of California, Davis, Sacramento, CA, United States; ^5^ Department of Medicine, Heersink School of Medicine, University of Alabama at Birmingham, Birmingham, AL, United States; ^6^ Center for Human Nutrition, Bloomberg School of Public Health, Johns Hopkins University, Baltimore, MD, United States; ^7^ Ministry of Health, Lusaka Zambia, Lusaka, Zambia; ^8^ Medical Faculty, Institute for Infectious Diseases, University of Bern, Bern, Switzerland

**Keywords:** social construction of ageing, Zambia ageing, non-communicable diseases, HIV, Zambia

## Abstract

**Objectives:**

We sought to understand the social construction of aging in a clinic-based population, with and without HIV, to address gaps in care for older individuals living with HIV in Zambia.

**Methods:**

Our exploratory qualitative study included 36 in-depth interviews with clinic clients and four focus group discussions with 36 professional and lay healthcare workers providing services to the clients. We identified themes based on social construction theory.

**Results:**

At the individual level, aging was multidimensional, perceived both as an achievement in the HIV era and as a period of cognitive, physical, and economic decline. In social interactions, older individuals were often stereotyped and treated as helpless, poor, and “witches.” Those living with HIV faced the additional stigma of being labeled as promiscuous. Some of the participants living without HIV refused to take daily medication for non-communicable diseases to avoid being mistaken for taking antiretroviral therapy for HIV. Older individuals wanted quality healthcare and family support to address the intersectional stigma of aging, poverty, and chronic illness.

**Conclusion:**

Multifaceted interventions are required to combat age-related prejudice, intersectional stigma, and discriminatory practices, particularly for people living with HIV.

## Introduction

The global introduction and scale-up of combination antiretroviral therapy (cART) have transformed HIV infection from a terminal illness to a chronic condition, resulting in an increasing number of people aging with HIV [1]. Aging people living with HIV often experience multiple non-communicable diseases (e.g., cardiovascular, renal, liver, bone disease and accelerated cognitive decline) and geriatric syndromes (e.g., frailty), potentially at higher rates and at younger ages than the general population [2–5]. In the context of HIV, UNAIDS has recognized people >50 years old as “older” [6]. In sub-Saharan Africa (SSA), an estimated 9.1 million people living with HIV will be aged 50 and above by 2040, that is one in four people living with HIV, a dramatic increase from the current one in seven [7]. People living with HIV are likely to face a vast array of health and social challenges commonly associated with aging in high-income settings, such as decreasing physical mobility, cognitive decline, increased risk of non-communicable diseases (NCDs), and polypharmacy [8]. At the same time, the intersectional stigma due to HIV, NCDs and aging along with related discrimination will create unique challenges for disease detection, monitoring, and the provision of health-related care necessary for a healthy, aging population [3, 4].

People’s perceptions of aging are socially constructed and evolve through individual, social, and organizational processes that reflect social and cultural norms [8–10]. These perceptions can lead to stereotypes that shape the behaviors and practices of both older individuals and those around them [8–10]. While historical age bias in global health policy is being addressed [11], healthcare facilities and public health initiatives often portray and treat older people as “weak” [2, 3, 5, 10, 11]. The majority of older people living with HIV in SSA experience fragmented health services for multi-morbidities and related polypharmacy, stigma, discrimination, and financial hardship, all of which interfere with their daily activities and family relationships [12]. Furthermore, perceptions of older people as asexual and inconsequential to society increase their risk for HIV and those living with HIV often report feelings of isolation, anxiety, and shame related to age, poverty, HIV, and multi-morbidities [2]. Understanding local perceptions of aging can provide important insights for the design of interventions that promote quality of life, which is a UNAIDS goal for people living with HIV in the delivery of healthcare [11, 13].

In response to the dual burden of HIV and NCDs, Zambia has begun the process of integrating HIV and NCD care by overlaying a mosaic of vertical and horizontal NCD and HIV programs at all levels of the healthcare system, accompanied by public awareness campaigns [14]. With an estimated 90% of people living with HIV in Zambia on cART, these services need to be expanded to meet their health needs as these individuals age [15] and take into consideration the culturally assigned meanings and attributes of aging and HIV [16, 17]. Understanding the cultural nuances around the concept of age and aging, societal expectations of older people, and their lived experiences from both community and healthcare worker perspectives during Zambia’s epidemiological transition can help to deliver culturally sensitive, integrated HIV and NCD services that are person-centered, a concept that has been proven to improve patient wellbeing and is being adapted by the Zambian Ministry of Health [18, 19]. This study aimed to explore the perceptions of aging and NCDs among people living with and without HIV who are attending outpatient clinics in Zambia, in addition to the health workers who care for them. Specifically, we sought to examine who is considered old, what attributes are associated with being old, how these perceptions affect the behavior and treatment of older people, and whether this is influenced by NCD and HIV status.

## Methods

From November 2020 to July 2021, we conducted an exploratory qualitative study with participants from a Clinic and a 1st Level Hospital, both characterized by a high HIV burden (>10,000 PLWH) and enrolled in the International Epidemiology Databases to Evaluate AIDS collaboration in Southern Africa (IeDEA-SA) study [20]. Ethical approval was provided by the University of Zambia Biomedical Research Ethics Committee (UNZABREC)-approval number 011-04-19.

We conducted 36 in-depth interviews (IDIs) with individuals from the IeDEA-SA cohort, with and without HIV, who presented for NCD screening including cardiometabolic conditions, substance use, and mental illness at study sites. We purposefully sampled for age (30–49 years, 50+) and HIV status: 1) newly diagnosed with HIV (<4 weeks on cART), 2) in longitudinal HIV care (>6 months on cART), and 3) tested HIV negative. Four focus group discussions (FGDs) with a convenience sample of 36 professional and lay healthcare workers (HCWs) providing care to people living with HIV were also conducted at these facilities to understand the conditions and experiences of managing multimorbidity. In the context of the COVID-19 epidemic, a convenience sample was appropriate to capture the views of professional and lay HCWs depending on their availability. Additionally, we did not collect demographic information on HCWs, as their interaction with us was minimized due to time costraints. Professional HCWs had medical education and provided clinical, nursing, cART, laboratory, and pharmacy services. Lay HCWs had no formal medical training; however, they offered critical, specialized services for which they had received specific training such as counseling and testing.

### Data Collection and Analysis

All participants gave written informed consent prior to participating in face-to-face, audio-recorded IDIs/FGDs in Bemba, Nyanja, and English as per their preference. Trained Research Assistants conducted interviews and discussions using semi-structured guides with open-ended questions (Supplementary Material S1, S2) [21]. All IDIs/FGDs were audio-recorded, translated directly into English, and checked for accuracy. Interviews were coded using reflexive thematic analysis [22] which consists of iterative coding and reflexive dialogue throughout. We then used deductive reasoning to categorize codes according to Johfre and Saperstein’s social construction theory [16] to interpret the shared functional meanings and experiences related to aging with HIV and NCDs. Through the lens of social construction theory, age and age groups can be understood as dynamic concepts that are both inputs and outcomes of social processes through which differentiation and inequalities are produced [16, 23]. Johfre and Saperstein [16] postulate that age has multiple dimensions at the individual level that can be measured by chronology, generation, life stage and experience, physical and cognitive function, socio-economic responsibilities, and cultural norms. These dimensions play out in social interactions, cultural practices, and national policies, such as the age of marriage, retirement, and social benefits.

## Results

A total of 36 individuals participated in the 45–60-min IDIs, of whom 61% were women, and 42% were >50 years old (Table 1). Twelve participants each were newly diagnosed with HIV, in longitudinal HIV care, and recently HIV-negative.

**TABLE 1:** Interview participant demographics (Lusaka, Zambia, 2020-2021).

Characteristic	Newly diagnosed with HIV (*n* = 12)	HIV longitudinal care (*n* = 12)	Recently tested HIV negative (*n* = 12)
Age category (years)			
30–49	8	3	9
≥50	4	9	3
Sex			
Male subjects	7	3	4
Female subjects	5	9	8

As a form of methodological and stakeholder triangulation, we also conducted four FGDs lasting 60–90 min with 36 healthcare workers.

We found three main themes—i) Aging, although a privilege, is usually associated with negative experiences and a loss of functional ability ii) Aging identity is mediated through social interactions and, iii) Social support and action are needed to mitigate ageism: participants’ ideas for better care. Ageism has been defined as discrimination against older adults based on age and includes their negative portrayal through stereotyping as sick, irrelevant and incompetent [11]. We used the definition provided by Anderson et al [24] to identify stigma in the data, i.e., “if and only if there is labeling, negative stereotyping, linguistic separation, and power asymmetry” that targets an entire group.

The major themes and sub-themes are presented in Table 2 along with Illustrative quotes in both Tables 2 and Supplementary Material S3.

**TABLE 2:** Themes and sub-themes categorized by social construction theory^a^ with illustrative quotes (Lusaka, Zambia, 2020-2021).

Measure—sub-theme	Illustrative quote
**Theme: Aging, although a privilege, is usually associated with negative experiences and loss of functional ability**	
Individual—experience	“*There is nothing good that comes with ageing*”
Generational—privilege	“*Growing old shows that you have taken good care of yourself*”
Physical—infantilization	“*They go back to being like a baby*”
Psychological—infantilization	“*They start to think, talk like they are a young child again*”
**Theme: Aging identity is mediated by social interactions**	
Life stage—poverty stigma	“*In the community, old people are viewed as desperate*”
Age stereotypes—stigma	“*I don’t know if they stop being a person when they get old*”
Age prejudice—HIV stigma	“*People that are aged and have HIV are not looked at in a good way*”
Cultural norms—respect	“*You must respect the aged*”
**Theme: Social support and action is needed to mitigate ageism:** participants’ ideas for better care	
Financial and physical support	“*I need someone to help me*”
Healthcare services	“*I would love to see that I am well attended to if I come to the clinic*”

^a^ Adapted from Johfre and Saperstein, Annu. Rev. Socio., 2023 [16].

The bold are themes as stated.

### Aging, Although a Privilege, Is Usually Associated With Negative Experiences and a Loss of Functional Ability

To participants from the IeDEA-SA cohort (hereafter referred to as laypersons, i.e., non-medically trained individuals) conceived of aging as 1) generational and 2) infantilization rather than a chronological passage of time. Overall, aging was perceived as regressive, weakening the individual and making them dependents due to reduced physical and cognitive capabilities. Older men and women stated that their bodies “*failed*” them as they aged and limited their activities. Those also living with HIV saw the long-term impact of HIV infection as an “*automatic*” progression to a state of frailty and susceptibility to infection. Nonetheless, aging was also seen as a privilege accorded to those who had taken “*good care*” of themselves and cART was viewed as an antidote to aging.

#### Age as Generational

Laypersons did not agree on the age at which a person may be considered old, offering a range of 50–100 years old. A few laypersons highlighted the privilege of longevity given the low life expectancy of people in Zambia (approximately 64.7 years according to the United Nations) [25], although they did not specify the age. A 31-year-old woman who screened negative for HIV observed that a long life was remarkable in Zambia, saying “*It is a good thing to grow old; it shows that you have taken good care of yourself; it’s not easy to a certain age in this generation, especially now with disease like HIV, a lot of people die young, but when you reach an old age, it is joyful to have reached that age.”* (Supplementary Material S3, quote 1)

#### Age as Infantilization

Despite viewing old age as an achievement, the general sentiment was that “*There is nothing good that comes with ageing*” due to changes in physical, psychological, social, and economic capabilities.

All laypersons, irrespective of age, gender or HIV status, had a negative outlook on the effects of aging and health conditions which they described as a loss of “*strength*” and “*energy*.” Aging was discussed by younger and older laypersons, both men and women, regardless of HIV status, as a negative process leading to health challenges, activity limitations and loss of physical abilities including physical weakness and pain, difficulty getting up and walking, and challenges with memory and cognitive functioning, reducing the cognitive and physical abilities of older people to those of young children (Supplementary Material S3, quotes 2–4).

Notably, some laypersons living with HIV, especially older men and younger women, shared their perceptions of increased vulnerability leading to frailty, which they described as skeletal issues that are inevitable “*if someone is HIV positive, as the years go by*,” because “*that is the only sad thing about people with HIV, the bones start deteriorating and a person cannot work well*” (Supplementary Material S3, quotes 5-6). Younger women, irrespective of HIV status, also felt that older people were susceptible to infections, which was likened to going “*back to being like a baby*” (Supplementary Material S3, quote 7). Laypersons, irrespective of age, gender or HIV status, and healthcare workers shared the belief that older people may experience immune system problems that could increase their vulnerability to illness and chronic health issues. This perceived vulnerability was referred to as “*weak blood*” in several interviews, to describe how the immune system becomes less effective with age (Supplementary Material S3, quote 8).

For these reasons, men and women living with HIV, regardless of age emphasized the importance of adherence to treatment as their cohort ages, citing it as a proactive approach to *“self-care”* and a safeguard against becoming weak (Supplementary Material S3, quote 9). In their opinion, poor adherence to HIV treatment, poor diet, and lifestyle choices like lack of exercise could lead to disease progression, and visible signs of illness, such as weight loss, physical weakness, and HIV-related deaths in older people living with HIV (Supplementary Material S3, quote 10).

### Identity in Old Age Is Mediated Through Social Interactions

Both laypersons and HCWs expressed a sense of concern and uncertainty about how older people are viewed and treated in society being both dehumanized and respected at the same time. They expressed persistent themes of intersectional stigma related to cultural beliefs and attitudes regarding poverty, aging, and living with HIV. Despite this, young people were expected to preserve their family image by being respectful to older people.

#### Stigma Associated With Aging and Poverty

Perceptions of physical and psychological weakness limited employment opportunities for older people to low-paying, low-intensity jobs. Elderly people were thought to be unemployable and engaged in restrictive roles due to their diminished physical and cognitive capabilities. Laypersons, irrespective of gender, age or HIV status, reported that employers tended to be skeptical about hiring older workers leaving them with “*menial*” job options such as night watchman (Supplementary Material S3, quote 11-12).

This discrimination fulfilled the common perception of older people as “*desperate, vulnerable people*” [Woman, 50 years, HIV+], who “*are seen like people with no money, just suffering to find some money, they live poorly*” [Woman, 43 years, HIV−]. Reduced or no income created challenges in managing NCDs and paying for required tests, medication, and lifestyle changes such as specific diets, both for the older person and their caregivers (Supplementary Material S3, quote 13-14). Those who had saved and managed to meet their own costs were considered to have been responsible in their youth.

#### Stigma Associated with Aging

Laypersons pointed out that older people were marginalized by the lack of recognition and attention for their presence and contributions, as though they “*stop being a person when they get old*” [Woman, 46 years, HIV−]. A 62-year-old woman living with HIV described feeling lonely and mentally ill due to social isolation, saying, “*When you are seated alone with no one to talk to you can die just from thinking*.” Younger women, irrespective of HIV status, emphasized the need for social interactions to prevent older people from feeling abandoned and neglected (Supplementary Material S3, quote 15).

Although not asked about witchcraft, almost every layperson spoke about the association of the elderly with witchcraft and other supernatural activities that influenced how they were perceived and treated in their communities. Almost every layperson spoke of elderly men and women being labeled “witches” as they were unrecognizable as their former selves and were an increased financial and social burden on communities (Supplementary Material S3, quote 16). Some younger laypersons acknowledged their reluctance and complexity in looking after older people, influenced by their association with witchcraft (Supplementary Material S3, quote 17). Some older laypersons complained that young people associated their aging with witchcraft practices and “*forgetting that there is no community without old people*” (Supplementary Material S3, quote 18).

#### HIV-Related Stigma

Older men living with HIV conveyed the difficulties of navigating old age and dealing with HIV stigma directed at them, with underlying judgments for “*contracting diseases for young people”* [Man, 70 years, HIV+]. A man, 51-year-old and living with HIV explained that, “*People that are aged and have HIV are not looked at in a good way, some people might say this person was promiscuous when they were young and that is how they got the HIV*.”

In FGDs, HCWs reported that some patients expressed concern about taking medication for NCDs such as diabetes due to potentially being mistakenly identified as people living with HIV or being likened to them because both diabetes and HIV are chronic conditions that require consistent treatment and frequent trips to the health facility (Supplementary Material S3, quote 19).

#### Respectful Age Relations

Despite this acknowledgment of stigma, younger women described cultural norms that require young people to show respect to older people in contrast to the negative beliefs described above. This respect was regarded as an important virtue symbolizing good manners and upbringing where “*Young people born in respectful families know how to respect elders*” [Woman, 43 years, HIV−] and with a middle-aged woman saying, “*To my side, I give respect to every old person that I see*” [Woman, 46 years, HIV−].

### Social Support and Action are Needed to Mitigate Ageism: Laypersons’ Ideas for Better Care

Laypersons, regardless of age, gender or HIV status wanted good healthcare services that would help older people regain their health and enable them to attend to their work and family responsibilities. Without these, they need family and social support due to loss of earning capacity and physical and cognitive abilities. They also said that they usually learned about NCDs after being diagnosed with one and along with HCWs, recommended awareness campaigns. HCWs including community volunteers recommended more training on NCDs and community-based screening for NCDs. In healthcare facilities, people living with HIV were more likely to be screened for NCDs than the general population.

#### Financial and Physical Support

Some older men and women highlighted feelings of vulnerability and increased dependence on their family due to reduced ability to meet their needs, with a 66-year-old HIV-negative man asking, “*Now that I am old, I ask who will give me food? I should be taken care of by my children because even the strength I had has reduced*.” Laypersons, irrespective of age, gender or HIV status, emphasized that as people age, they often require assistance with daily activities, care, and support as they may not “*stand*” on their own. Both they and the HCWs discussed the central role of family and social support in caring for and supporting older people, with a 46-year-old HIV-negative woman saying, “*The aged need home care. Old people need someone to take care of them, bath them and feed them*.”

#### Healthcare Services

Laypersons irrespective of age, gender or HIV status, emphasized the importance of having a comprehensive and positive healthcare experience as an older person (Supplementary Material S3, quote 20). Some laypersons, especially older men, expressed a desire to recover from the illnesses they had been diagnosed with to regain their health, return to work, and lead “*a good life … that is in the middle, in a normal way that a person is supposed to live on this earth”* (Supplementary Material S3, quote 21).

Laypersons irrespective of age, gender or HIV status, said that “*there is lack of information about NCDs in the community*” due to a lack of awareness campaigns such as those for “*common disease like HIV, cholera, typhoid*” [SIC] [Man, 34 years, HIV-], and that, “*Here in Zambia, … We normally come to know NCDs when you are sick*” [Woman, 51 years, HIV+]. In FGDs, HCWs corroborated the finding about lack of information and left room for questions regarding NCDs; for example, poor people who believed that diabetes primarily affects wealthy individuals, questioned their diagnosis asking “*How did this come to be? I don’t have money and this disease is for people who are rich*” [Participant 3, FGD 4, lay HCW]. Others, they said, might resort to herbal treatments due to perceptions such as “*when you start taking BP drugs, that’s when BP becomes worse*” and the association of lifelong treatment with HIV (Supplementary Material S3, quote 22).

HCWs reported screening people living with HIV for NCDs such as cervical cancer on their own initiative and that “*there are no people coming just for NCD screening only*” [Participant 4, FGD 1, professional health worker]. They suggested community-based testing as “*a way of bringing health services closer and people will be eager to check for BP, diabetes*” [Participant 7, FGD 1 professional health worker], based on the success of community-based HIV testing and treatment strategies. With this proactive approach, they thought that care would not be solely dependent on people attending healthcare facilities. However, they reported being trained in HIV and not in managing the unique health needs of individuals with multiple chronic conditions. They recommended training in this area for all HCWs saying, “*We really need this training in order to manage these diseases in this day and age. And the training should not just be for Clinical Officers but all health provider*s” [Participant 6, FGD 3, professional health worker]. Lay HCWs emphasized their desire to be included in training to equip them with the knowledge and skills necessary to identify individuals with NCDs within the community (clinic catchment area) and assist them in seeking appropriate medical care, saying, “*You know information is power … people perish because of lack of knowledge and, also ignorance is very expensive*” (Supplementary Material S3, quotes 23-24).

## Discussion

This study examined the concept of aging in the context of HIV and NCDs, gathering perspectives from both people living with and without HIV in SSA. We found a strong perception of aging as a period of functional decline and ill health that strains social relationships and reduces the sense of wellbeing of older people. Participants respected old age, especially in the HIV era and were aware of the emotional distress caused to older people when they were ignored, stigmatized, and treated as a financial and physical burden. Older people living with HIV were labeled as promiscuous and others on lifelong medication were suspected of taking antiretrovirals. Communities stigmatized older people who were poor. Although older people wanted “*a good life*” marked by physical and financial independence, screening and management of chronic conditions at the clinic were limited due to the low level of training and comfort of health workers in managing NCDs.

Prevalent societal stereotypes that associate aging with decline, loss of strength and reduced energy fuel negative perceptions of older people that impact how they view their own capabilities and self-worth. These stereotypes harbor contradictory societal attitudes that both dehumanize and respect older people, a duality found in both urban and rural Zambia [17, 26, 27]. In our study, as people grew older, they were considered unrecognizable due to their reduced functional capacity leading to their being suspected of practicing witchcraft. This association in the context of high-density, resource-poor residential areas may encompass 1) a desire for longevity and power that eludes many; 2) a fear of life-threatening conditions and, in the larger community, 3) socio-economic competition [28]. In practice, this has allowed families to intermittently pass on caring responsibilities to other relatives, contrary to expectations of reciprocity from family and church members for contributions made in earlier years [17, 27]. Thus, fears of cognitive decline, physical weakness, and illness arise from complex physical, mental, emotional, and social and cultural dimensions that encompass health and its importance in maintaining “*a good life*” for older people.

Conflicting social and cultural beliefs impact intergenerational relationships, which are already complicated by altered family dynamics due to the loss of the “middle generation” to HIV [17]. The intersectional stigma experienced by older individuals related to aging and poverty within their own families can lead to increased vulnerability and isolation [17, 26–28]. Perceptions of increased vulnerability to frailty and infection among people living with HIV add another layer of concern about aging. HIV stigma and the association of NCD treatment regimens with antiretrovirals can lead to hesitancy in seeking medical treatment and in adhering to prescribed regimens (e.g., anti-hypertensive medications) [29]. Limited job options add to the financial strain of managing chronic conditions and suggest age-related discrimination in the workplace in addition to older people taking on the assumption that they are not capable of performing work [27]. The Zambian Ministry of Health promotes family and social support due to loss of functional and earning capacity, and supports networks for people living with chronic conditions [30]. However, intersectional stigma requires intersectional discrimination laws and multisectoral policies that recognize the different dimensions of aging in their full context to make transformative changes [29, 31]. For instance, older people may experience unemployment, job loss or lower wages as a result of multiple social identities, such as “living with a chronic condition,” “disabled,” and “poor.” Discrimination laws will need to ensure that these aspects along with, for example, gender, sexuality, and age, are treated synergistically. Policies will need to mandate changes, for example, in accessibility, urban planning, and social and health services to enable olderpersons to have functional ability and a sense of well-being.

The desire of people living with HIV for good healthcare services that promote recovery and enable them to fulfill their work and family responsibilities underscores the importance of accessible and effective healthcare systems, revised labor policies, and the need for improved intergenerational relations. The perceived vulnerability and expectation of reduced functioning due to aging and HIV may motivate some to adhere to treatment, seek a healthy lifestyle and avoid disease progression [32]. However, interpersonal dialogue is needed to identify and address individual beliefs that delay recognition and acceptance of diagnosis and treatment, with detrimental effects due to untreated HIV and NCDs [33]. While decentralized and differentiated HIV service delivery in Zambia has made care more available, accessible, affordable and accommodating, older people may find services that separate them from younger people and are provided by peers more acceptable. In Zambia, health services do not directly serve older people, i.e., there are no age-specific clinics except for children and adolescents. Nonetheless, women may be more likely than men to access “blameless” testing due to their caregiving role including for family members living with HIV [34]. Finally, older people seeking care may also need help navigating the National Health Insurance Scheme and Social Cash Transfer programs to mitigate financial losses due to age and ill health [35].

Frey *et a*l [29] offer many suggestions for HIV primary healthcare clinics to better serve older people living with HIV including involving them in clinical research and designing clinical programs, focusing on optimizing ability, creating guidelines and multi-disciplinary teams to manage multimorbidities, and coupling age and HIV-related social services. Lay healthcare worker willingness and the call for community-based screening activities, provide an opportunity to consider solutions such as healthcare worker-facilitated screening incorporating mHealth, for example, mobile applications to guide screening procedures, capture screening/rapid test results, and link to telehealth, including for counseling and mental health interventions [36–38]. People living with HIV in our study also suggested self-care, providing examples of maintenance behaviors such as diet, exercise, and taking cART as prescribed. These maintenance behaviors should be combined with monitoring, for example, viral loads, and managing signs of ill health, such as engagement with the healthcare system [39]. No substance use, adequate sleep, and family support can also contribute to healthy aging [40]. In the Zambian context, engagement with family, social groups, and the church can increase social connectedness and support in times of need [17, 18, 27]. Engaging with family may mean handing over caregiving responsibilities if they are a source of financial and physical strain and also reminding younger family members of cultural expectations of reciprocity [27, 41]. In Zambia, church and social groups, such as neighborhood health committees, water committees, safe motherhood action groups, and treatment support groups, provide opportunities for older people to interact, teach and learn from their peers and to interact with younger people, share their experiences and stay up to date with new technologies [42]. Joining existing coalition groups such as the Treatment Action Group can also be an avenue for articulating needs and advocating for improved policies and programs [2].

Older people, including married couples, may also experience additional stressors due to differences in sexuality; studies in SSA suggest that while more than half the men reported being sexually active after the age of 65 and well into their 80s, fewer women claimed to be sexually active after age 45 [2]. This increases the possibility of polygyny, continued HIV transmission, and late HIV detection due to a low perceived risk of transmission [2]. Similarly, older people may overestimate their risk but not seek testing services due to embarrassment, adding to their stress [42–44]. In the absence of health programs to address concerns about changes in sexual function, churches and traditional teaching mechanisms in Zambia may prove valuable in holding what are locally called “conferences” on physical, sexual, financial and spiritual wellbeing. These could address communication and conflict resolution in couples, sexual health in HIV, sexual negotiation, instrumental and emotional support, enhancing family health with limited resources, intimate partner violence, and substance use [45].

The major strength of this study was its integration into a robust HIV-NCD project; thus, participants had first-hand experience of being screened and treated for NCDs in the same health facilities, in the presence or absence of HIV infection. Despite this, we found limited biomedical knowledge about NCDs and treatment among laypersons. Our study also has limitations, notably that it was facility-based, which tended to exclude those who were unable to access healthcare. It was also undertaken in an urban area; therefore, rural experiences may differ. COVID-19 restrictions did not allow for observations and policymakers were busy ensuring continuity of care services: this would have provided a richer context. Also, the results reflect the views of younger people (60%) although at 40%, older people are overrepresented in our sample selection compared to the HIV population (14%). Finally, we began data collection during the implementation of the current national health strategic plan [30], which limited the incorporation of specific knowledge and practice recommendations arising from the interviews into policy. Nonetheless, this study provides key insights into social and cultural norms that feed into and are fed by perceptions of aging and older people. As a qualitative study, our findings are based on a small, non-generalizable sample. However, our use of IDIs with both people living with and without HIV and of FGDs with both professional and lay healthcare workers, yields deep insights that can be further explored in settings seeking to integrate NCD and HIV services.

### Conclusion

The complex and multidimensional nature of how older people are perceived and treated in Zambia requires multi-faceted interventions to combat ageism, intersectional stigma, and discriminatory practices to improve their overall wellbeing, preserve their dignity and promote their social inclusion, particularly for those living with chronic diseases such as HIV. As ageism operates at different levels, cross-cutting social, economic, and health interventions are needed; for example, promotion of intergenerational communication and respect at the community level, health promotion campaigns at health system level, and addressing job discrimination at the policy level.

## References

[1] Kharsany AB Karim QA. HIV Infection and AIDS in Sub-Saharan Africa: Current Status, Challenges and Opportunities. Open AIDS J (2016) 10:34–48. 10.2174/1874613601610010034 27347270 PMC4893541

[2] Finkelstein RK Gonsalves GS Brennan-Ing M Brennan-Ing M Porter KE Kaufman JE Aging With HIV in Sub-Saharan Africa: Health and Psychosocial Perspectives. Cham: Springer International Publishing AG (2022). 10.1007/978-3-030-96368-2

[3] Lazarus JV Safreed-Harmon K Kamarulzaman A Anderson J Leite RB Behrens G Consensus Statement on the Role of Health Systems in Advancing the Long-Term Well-Being of People Living With HIV. Nat Commun (2021) 12(1):4450. 10.1038/s41467-021-24673-w 34272399 PMC8285468

[4] Coetzee L Bogler L De Neve JW Bärnighausen T Geldsetzer P Vollmer S. HIV, Antiretroviral Therapy and Non‐Communicable Diseases in Sub‐Saharan Africa: Empirical Evidence From 44 Countries Over the Period 2000 to 2016. J Int AIDS Soc (2019) 22(7):e25364. 10.1002/jia2.25364 31353831 PMC6661400

[5] Rodes B Cadinanos J Esteban-Cantos A Rodríguez-Centeno J Arribas JR. Ageing With HIV: Challenges and Biomarkers. EBioMedicine (2022) 77:103896. 10.1016/j.ebiom.2022.103896 35228014 PMC8889090

[6] UNAIDS. 2006 Report on the Global AIDS Epidemic. Geneva: Switzerland: UNAIDS (2006).

[7] Negin J Mills EJ Bärnighausen T. Aging With HIV in Africa: The Challenges of Living Longer. AIDS (London, England) (2012) 26(0 1):S1–5. 10.1097/QAD.0b013e3283560f54 22713477 PMC4017661

[8] Guaraldi G Malagoli A Calcagno A Mussi C Celesia BM Carli F The Increasing Burden and Complexity of Multi-Morbidity and Polypharmacy in Geriatric HIV Patients: A Cross Sectional Study of People Aged 65–74 Years and More Than 75 Years. BMC Geriatr (2018) 18:99–0. 10.1186/s12877-018-0789-0 29678160 PMC5910563

[9] Westerhof GJ Nehrkorn-Bailey AM Tseng HY Brothers A Siebert JS Wurm S Longitudinal Effects of Subjective Aging on Health and Longevity: An Updated Meta-Analysis. Psychol Aging (2023) 38(3):147–66. 10.1037/pag0000737 36972091 PMC10192139

[10] Kelly G Mrengqwa L Geffen L. “They Don’t Care about Us”: Older People’s Experiences of Primary Healthcare in Cape Town, South Africa. BMC Geriatr (2019) 19(1):98–4. 10.1186/s12877-019-1116-0 30947709 PMC6449977

[11] Ng R Chow TY Yang W. Culture Linked to Increasing Ageism During COVID-19: Evidence From a 10-Billion-Word Corpus Across 20 Countries. The Journals Gerontol Ser B (2021) 76(9):1808–16. 10.1093/geronb/gbab057 PMC8083600 33786581

[12] Yang Z Zhu Z Lizarondo L Xing W Han S Hu H Experience of Chronic Noncommunicable Disease in People Living With HIV: A Systematic Review and Meta-Aggregation of Qualitative Studies. BMC Public Health (2021) 21(1):1651–9. 10.1186/s12889-021-11698-5 34507576 PMC8431942

[13] Harris TG Rabkin M El-Sadr WM. Achieving the Fourth 90: Healthy Aging for People Living With HIV. AIDS (London, England) (2018) 32(12):1563–9. 10.1097/QAD.0000000000001870 29762172 PMC6082594

[14] Richter P Aslam M Kostova D Lasu AA Vliet GV Courtney LP The Case for Integrating Health Systems to Manage Noncommunicable and Infectious Diseases in Low-And Middle-Income Countries: Lessons Learned From Zambia. Health Security (2022) 20(4):286–97. 10.1089/hs.2022.0016 35904943

[15] Kietrys D Myezwa H Galantino ML Parrott JS Davis T Levin T Functional Limitations and Disability in Persons Living With HIV in South Africa and United States: Similarities and Differences. J Int Assoc Providers AIDS Care (Jiapac) (2019) 18:2325958219850558. 10.1177/2325958219850558 PMC6748470 31109225

[16] Johfre S Saperstein A. The Social Construction of Age: Concepts and Measurement. Annu Rev Sociol (2023) 49:339–58. 10.1146/annurev-soc-031021-121020

[17] Kabelenga I. Perceptions of Community Leaders About Normative Understandings of Good Family Care of Older People in Rural and Urban Zambia. Int J Care Caring (2023) 7(2):326–42. 10.1332/239788221X16770625208518

[18] Mwamba C Beres LK Mukamba N Jere L Foloko M Lumbo K Provider Perspectives on Patient‐Centredness: Participatory Formative Research and Rapid Analysis Methods to Inform the Design and Implementation of a Facility‐Based HIV Care Improvement Intervention in Zambia. J Int AIDS Soc (2023) 26(S1):e26114. 10.1002/jia2.26114 37408458 PMC10323320

[19] CIDRZ. Person Centered Care (PCC) to Boost Health Care Services to Recommended Standard Treatment Guidelines (2023). Available from: https://www.cidrz.org/2023/02/08/person-centered-care-pcc-to-boost-health-care-services-to-recommended-standard-treatment-guidelines (Accessed February 12, 2024).

[20] Chihota BV Mandiriri A Shamu T Muula G Nyamutowa H Taderera C Metabolic Syndrome Among Treatment‐Naïve People Living With and Without HIV in Zambia and Zimbabwe: A Cross‐Sectional Analysis. J Int AIDS Soc (2022) 25(12):e26047. 10.1002/jia2.26047 36522287 PMC9755006

[21] Renjith V Yesodharan R Noronha JA Ladd E George A. Qualitative Methods in Health Care Research. Int J Prev Med (2021) 12:20. PMID: 34084317; PMCID: PMC8106287. 10.4103/ijpvm.IJPVM_321_19 34084317 PMC8106287

[22] Byrne D. A Worked Example of Braun and Clarke’s Approach to Reflexive Thematic Analysis. Qual Quantity (2022) 56(3):1391–412. 10.1007/s11135-021-01182-y

[23] Powell JL Hendricks J. The Sociological Construction of Ageing: Lessons for Theorising. Int J Sociol Soc Pol (2009) 29(1/2):84–94. 10.1108/01443330910934745

[24] Andersen MM Varga S Folker AP. On the Definition of Stigma. J Eval Clin Pract (2022) 28(5):847–53. 10.1111/jep.13684 35462457 PMC9790447

[25] United Nations. World Population Prospects (2023). Available from: https://www.macrotrends.net/countries/ZMB/zambia/life-expectancy#:∼:text=The%20current%20life%20expectancy%20for,a%200.45%25%20increase%20from%202021 (Accessed September 7, 2023).

[26] Chirwa M Kalinda R. Challenges of the Elderly in Zambia. A Systematic Review Study. Eur Scientific J (2016) 12(2):351. 10.19044/esj.2016.v12n2p351

[27] Dhónaill CN. Positive and Negative Perceptions of Ageing Identity Compared to Lived Experience: A Comparative Study of Older People in Zambia and Northern Ireland. Comp Sociol (2015) 14(3):402–27. 10.1163/15691330-11341351

[28] Mildnerová K. The Modern Forms of Witchcraft in Zambia: An Analysis of Local Witchcraft Narratives in Urban Settings of Lusaka. Relio (2016) 24:19–51.

[29] Frey E Johnston CD Siegler EL. Treatment Regimens and Care Models for Older Patients Living With HIV: Are We Doing Enough? HIV/AIDS-Research Palliat Care (2023) 31:191–208. 10.2147/HIV.S311613 PMC10155713 37153650

[30] MOH. 2022 – 2026 National Health Strategic Plan: “Towards Attainment of Quality Universal Health Coverage Through Decentralisation (2023). Available from: https://www.moh.gov.zm/wp-content/uploads/2023/02/National-Health-Stratergic-Plan-for-Zambia-2022-to-2026-revised-February-2023-lower-resolution.pdf (Accessed August 18, 2023).

[31] Atrey S. Intersectional Discrimination. USA: Oxford University Press (2019).

[32] Hagger MS Orbell S. The Common Sense Model of Illness Self-Regulation: A Conceptual Review and Proposed Extended Model. Health Psychol Rev (2022) 16(3):347–77. 10.1080/17437199.2021.1878050 33461402

[33] Mabuto T Charalambous S Hoffmann CJ. Effective Interpersonal Health Communication for Linkage to Care After HIV Diagnosis in South Africa. J Acquired Immune Deficiency Syndromes (2017) 74(1):S23–S28. 10.1097/QAI.0000000000001205 PMC5147038 27930608

[34] Mojola SA Angotti N. Sometimes It Is Not About Men’: Gendered and Generational Discourses of Caregiving HIV Transmission in a Rural South African Setting. Glob Public Health (2022) 17(12):4043–55. 10.1080/17441692.2019.1606265 31014204 PMC6812629

[35] Mazimba Z Muwowo EC Chilufya KM Malumani M. The National Health Insurance Scheme (NHIS) and the Attainment of Universal Health Coverage in Zambia. Rw Public Health Bull (2023) 4(1):53–6.

[36] LeBan K Kok M Perry HB. Community Health Workers at the Dawn of a New Era: CHWs’ Relationships With the Health System and Communities. Health Res Pol Syst (2021) 19(3):1–9. 10.1186/s12961-021-00756-4 PMC8506091 34641902

[37] Naslund JA Shidhaye R Patel V. Digital Technology for Building Capacity of Non-Specialist Health Workers for Task-Sharing and Scaling up Mental Health Care Globally. Harv Rev Psychiatry (2019) 27(3):181–92. 10.1097/HRP.0000000000000217 30958400 PMC6517068

[38] Ma KS. Screening Programs Incorporating Big Data Analytics. Big Data Analytics Healthc (2022) 2022:313–27. 10.1016/B978-0-323-91907-4.00023-6

[39] Matarese M Lommi M De Marinis MG Riegel B. A Systematic Review and Integration of Concept Analyses of Self‐Care and Related Concepts. J Nurs Scholarship (2018) 50(3):296–305. 10.1111/jnu.12385 29645402

[40] Omotara BA Yahya SJ Wudiri Z Amodu MO Bimba JS Unyime J. Assessment of the Determinants of Healthy Ageing Among the Rural Elderly of North-Eastern Nigeria. Health (2015) 7(06):754–64. 10.4236/health.2015.76090

[41] De Klerk J Moyer E. “A Body Like a Baby”: Social Self-Care Among Older People With Chronic HIV in Mombasa. Med Anthropol (2017) 36(4):305–18. 10.1080/01459740.2016.1235573 27644708

[42] Naah FL Njong AM Kimengsi JN. Determinants of Active and Healthy Ageing in Sub-Saharan Africa: Evidence From Cameroon. Int J Environ Res Public Health (2020) 17(9):3038. 10.3390/ijerph17093038 32349334 PMC7246554

[43] Johnson C Kumwenda M Meghji J Choko AT Phiri M Hatzold K Too Old to Test?’: A Life Course Approach to HIV-Related Risk and Self-Testing Among Midlife-Older Adults in Malawi. BMC Public Health (2021) 21:1–3. 10.1186/s12889-021-10573-7 33812381 PMC8019342

[44] van Empel E de Vlieg RA Montana L Gómez-Olivé FX Kahn K Tollman S Older Adults Vastly Overestimate Both HIV Acquisition Risk and HIV Prevalence in Rural South Africa. Arch Sex Behav (2021) 50:3257–76. 10.1007/s10508-021-01982-1 34599468 PMC8563552

[45] Hampanda K Matenga TF Nkwemu S Shankalala P Chi BH Darbes LA Designing a Couple-Based Relationship Strengthening and Health Enhancing Intervention for Pregnant Women Living With HIV and Their Male Partners in Zambia: Interview Findings From the Target Community. Soc Sci Med (2021) 283:114029. 10.1016/j.socscimed.2021.114029 34242890 PMC10790566

