# Supplementary material for: The Social Construction of Aging Among a Clinic-Based Population and Their Healthcare Workers in Zambia
Source: Int J Public Health. 2024 Apr 22;69:1606607. doi: 10.3389/ijph.2024.1606607 (PMC11070831; doi:10.3389/ijph.2024.1606607)
Supplement: Supplementary file 1 [file DataSheet3.docx]

**Supplementary Material S3. Ageing and Comorbidities among HIV infected and uninfected adults attending primary care clinics in Lusaka, Zambia (2020-2021)**

**Supporting Quotes in the Order Cited in Text**

| # | Quote | Speaker |
| --- | --- | --- |
| 1 | ‘The gains are there, it is a good thing to grow old, it shows that you have taken good care of yourself. It’s not easy to reach a certain age in this generation especially now with a disease like HIV, a lot of people die young, but when you reach an old age, it is joyful to reach that age.’ | Female, 31yrs, HIV- |
| 2 | ‘Like for me, I have aged, I have no strength to work, I can’t even walk long distances, I just walk short distances, I have no energy.’ | Male, 66yrs, HIV- |
| 3 | ‘Some people need to be carried to bed, carried to the toilet, so there are no good things, in my opinion.’ | Male, 51yrs, HIV+ |
| 4 | ‘Many people develop problems with their memory. They start to think, talk like they are a young child again then that means that the brain has become weak.’ | Male, 47yrs, HIV- |
| 5 | Frailty, the way I understand, let’s say if someone is HIV positive, as the years go by, that person will automatically begin to be frail.’ | Female, 33yrs, HIV_+_ |
| 6 | ‘The bones start deteriorating that is the only sad thing about people with HIV the bones start deteriorating and a person cannot work well.’ | Male, 70yrs, HIV+ |
| 7 | ‘Someone who is ageing usually gets sick time to time because diseases easily get inside them. I can just say they go back to being like a baby because a baby easily gets sick.’ [Female, 32yrs, HIV+] | Female, 32yrs, HIV+ |
| 8 | ‘The problem that is there when a person is old is that the blood is weak, so they have problems with diseases. Even after a short period of time, it comes again because the blood is not okay and its different from those that are young (…). When I say the blood is weak, I mean contracting of diseases is quicker.’ | Male, 51yrs, HIV+ |
| 9 | ‘Even though they are old, they survive and they just look okay, and the health is fine. Taking care of oneself is taking the same ART medication boosts.’ | Male, 45yrs, HIV+ |
| 10 | ‘So, what is there is that, if you are found with HIV, you cannot age faster because you will be taking some tablets (ARVs), but those who do not take the medicine are those who you see losing weight and getting weak until they die.’ | Male, 64yrs, HIV+ |
| 11 | ‘You can’t find work when you grow older as power reduces, so old people need help fending for their livelihoods.’ | Female, 56yrs, HIV+ |
| 12 | ‘Jobs offered to old people are very few because they will do not perform according to the way certain things are supposed to be done, so employers would rather just consider older people to do low jobs like to be security guards.’ | Female, 46yrs, HIV+ |
| 13 | ‘Challenges increase as you grow. The challenges that we have, for those of us with diabetes is that we want to eat anytime, I have discovered that diabetes is hunger. Diabetes can kill. If you don’t follow its conditions, people die. But you find you don’t have food.’ | Female, 62yrs, HIV- |
| 14 | ‘It comes to relatives not having the money for medical tests as the government cannot afford providing such free to each and every person. Relatives may also not have money to buy medicine. The family may also not have money to buy food for that particular person because when somebody is sick, he or she needs to eat and to be taken care of very well as a patient.’ | Female, 51yrs, HIV+ |
| 15 | ‘As a person is aging, they need someone to be there that can talk to them otherwise they start having worries and thinking people have abandoned them and do not want them.’ | Female, 32yrs, HIV+ |
| 16 | ‘The reason why they are perceived to be witches is that when a person grows old, their actions are strange, they just do anything. They might pee on themselves. They easily get upset, they would refuse to eat, they would complain that ‘I don’t know how you perceive me.’ Those who have not stayed with an old person would say that they are witches when they are not.’ | Female, 36yrs, HIV- |
| 17 | ‘My friend, no matter how old a person gets, helping them is very difficult. It’s difficult even when it’s your parent and you will want to take them elsewhere maybe to live with their younger sibling because you cannot stay with an old person every day. They do many witchcraft things.’ | Male, 45yrs, HIV+ |
| 18 | ‘You know with certain young people, you find that they never grew with their parents or maybe they’ve never met their grandparents, now coming in our community when they see us, they just go like- these are the same ones bewitching me, these same old women, they are witches, she was in my dreams, forgetting that there is no community without old people.’ | Female, 62yrs, HIV- |
| 19 | ‘Some clients say that once you start taking medication for diabetes then they (community members) start comparing themselves with people living with HIV. That’s the fear because diabetes is a chronic illness and it being a chronic illness, you have to take the medicine on a daily basis to maintain the sugar levels. So they fear when they start taking the diabetes drugs they will be regarded as HIV positive and yet some are negative.’ | Participant 2, FGD 3 professional HCW |
| 20 | ‘For an old person like me, ok, I would love to see that I am well attended to if I come to the clinic, and the complications that I am undergoing, are lessened’ | Male, 60yrs, HIV+ |
| 21 | ‘‘I want to have a good life. To be able to do my business. I do not want to get too rich and at the same time I do not want to suffer too much. I just want a life that is in the middle in a normal way that a person is supposed to live on this earth’ | Male, 60yrs, HIV+ |
| 22 | ‘In the community, they say that when you start taking BP drugs, that’s when BP becomes worse, you see? And then they are saying, ... I should start taking BP drugs like I am taking ARVs. So, all that brings people backwards, they will start taking herbs so that BP can go down. | Participant 2, FGD 3, professional HCW |
| 23 | ‘The training (NCD) is for doctors and nurses only. We also need to be trained with a full package so that when we go into the community, we should be able to identify people with NCDs and help them so that they seek medical attention’ | Participant 3, FGD 2, lay HCW |
| 24 | 'Information is needed in the community and if we have those trainings and then we can go out in the community to educate them. You know information is power ... people perish because of lack of knowledge and, also ignorance is very expensive.’ | Participant 6, FGD 4 lay HCW |
