# Supplementary material for: The Social Construction of Aging Among a Clinic-Based Population and Their Healthcare Workers in Zambia
Source: Int J Public Health. 2024 Apr 22;69:1606607. doi: 10.3389/ijph.2024.1606607 (PMC11070831; doi:10.3389/ijph.2024.1606607)
Supplement: Supplementary file 2 [file DataSheet2.docx]

**Supplementary Material S2. Ageing and Comorbidities among HIV infected and uninfected adults attending primary care clinics in Lusaka, Zambia (2020-2021)**

**Focus Group Discussion Guide**

**Target Audience: Healthcare Workers**

**Recruitment**

Hello. How are you? My name is ____. As discussed, the Centre for Infectious Disease Research in Zambia (CIDRZ) is recruiting health care workers to participate in a focus group discussion on noncommunicable diseases and multimorbidity related patient care at your health facility. Participation is completely voluntary and your decision to participate or not will not affect your job at this facility. Are you potentially interested in participating? If so, I will give you additional information before you decide to participate. [If yes, proceed with ICF. If no, thank them for their time.]

**Staffing**

Facilitator

Co-facilitator

Notetaker

**Materials**

Flip chart/paper

Markers

2 digital voice recorders, spare batteries

Number tags (We supply number tags 1-10 and people use that to refer to self and others)

FGD guide

Consent/information sheets

Reimbursement form

Participant list/enrolment log

Pens

**Introduction**

Good morning/afternoon. Thank you for taking the time to participate in this focus group discussion. My name is ______. This is my colleague_____[note taker name] _____________.

**Purpose of focus group**

For reasons of confidentiality, we will not be introducing ourselves to each other – even if we know each other, we ask that no names be used – but rather the numbers assigned to you. Also, we ask for shared confidentiality, anything said in this room should not be shared outside.

Before we begin, I want to tell you a little bit about today’s discussion and what we are going to do here today.

We want to understand your experiences with managing noncommunicable diseases (NCDs) and multimorbidity among adult clients seeking care at your facilities. Ultimately, we want to understand the context of NCD and multimorbidity management in Zambia. This is one of 6 focus groups that we will be conducting with health care providers at different public health facilities. The information from today’s group will help us to develop ways to improve NCD diagnosis and management in the community and at this facility. You have been invited here because we want a wide range of perspectives on NCD services and ways to improve these. There is no right or wrong answer – we simply want to understand the reality of the community and facility.

This group is scheduled to last approximately 45 minutes. We will be recording the discussion so that we can check our understanding. The notes will help us in case the recording fails.

Does anyone have any questions so far?

If not, then we would like to begin.

|  | **Questions** | **Probes** |
| --- | --- | --- |
| **Introduction** | Can you explain your role in the management of noncommunicable diseases?  (*example-checking blood pressure, measuring height/ weight, adherence counselling)* |  |
|  | What brings most adults to your clinic/hospital? | - Does this differ between men and women? |
| **Knowledge on multimorbidity and NCDs** | What noncommunicable diseases (NCDs) do you know?  Tell me about any guidelines or materials you have heard of for diagnosing or treating the diseases you mentioned above?  What training have you received in managing NCDs and multimorbidity?  Do you feel like you have had all the training you need to treat NCDs and multimorbidity or do you wish you had more training in some areas?  What gaps in knowledge or misconceptions about NCDs do you commonly see among adults coming for NCD screening and testing? | - Does this differ between those who are HIV positive and those who are HIV negative?  -By who and when?  - In what areas do you feel you need more training?  -How would you raise more awareness about these gaps? |
| **Management and decision-making around NCD services** | How do you manage patients with multimorbidity/NCDs?  What tools and infrastructure do you have to manage NCDs/multimorbidity? | - Does this differ between those who are HIV-positive and HIV-negative?  - What treatment and care are they given?  - diagnostic equipment i.e. BP machines, glucose monitors, X-rays?  - space/dedicated departments? |
| **Barriers and Facilitators to NCD** **services** | What challenges do you face with managing multimorbidity/NCDs?  Are there things that could help you personally assist patients better? Things that the clinic/ government could do? | - Does this differ between men and women? How?  -Does this differ between HIV positives and HIV negatives |
| **Experiences- Reflections and recommendations** | What would be the best way to reach community members with **information** about where to access NCD testing and treatment services?  Are there other activities that you think the clinic or facility should be doing that they aren’t currently, and should be a priority? | 1. Screening **all** older patients coming to clinic even if there is no clinical indication?  2. Offering comprehensive care packages dedicated to managing multimorbidity |
| **Wrap Up** | We have talked about many things related to NCDs and multimorbidity. Are there any final thoughts you would like to share with me? Do you have any questions? |  |
