# Supplementary material for: The Social Construction of Aging Among a Clinic-Based Population and Their Healthcare Workers in Zambia
Source: Int J Public Health. 2024 Apr 22;69:1606607. doi: 10.3389/ijph.2024.1606607 (PMC11070831; doi:10.3389/ijph.2024.1606607)
Supplement: Supplementary file 3 [file DataSheet1.docx]

**Supplementary Material S1. Ageing and Comorbidities among HIV infected and uninfected adults attending primary care clinics in Lusaka, Zambia (2020-2021)**

**In-Depth Interview Guide**

**Target Audience: IeDEA NCD/SRN Participants (Adults 30 years and older)**

| **Welcome Remarks**   - Thank you for agreeing to be part of this interview. - My name is ____ from the Centre for Infectious Disease Research in Zambia (CIDRZ). - We will be asking you questions around your knowledge, perceptions and beliefs with regards to ageing and multimorbidity. - What we will discuss today will be kept confidential. This means that we will not disclose your name or personal details as we use this information. - We will be recording this interview using this voice recorder. This is because we will not be able to remember everything that was discussed in this interview. Please let us know if this is ok with you. |
| --- |

1. **Knowledge on Mutimorbidity (NCDs) and Ageing**

*I would like to ask you on your knowledge and understanding of illnesses and ageing*

1. What do people your age know about Noncommunicable diseases (examples like Diabetes, Hypertension, Kidney disease, Liver disease)?
2. Can you tell me about some of the challenges you think someone might have when dealing with more than one/ multiple illnesses (multimorbidity) like those mentioned above?
3. How do you think these challenges are influenced/ determined (either positively or negatively) by ageing?
4. **Perceptions on Ageing and Geriatric Syndromes**

*Now I would like us to talk about ageing*

- - - 1. Can you describe what a typical old person is like?

***Probe***

- Age
- Life Circumstance
- State of being
- Society rank
- Employment status
  - - 1. When you think about an old person how do they look physically?

1. How would you describe their mental or psychological state?

1. How do people in society or those in your community view an old person (the one

you just described)?

***Probe***

- Socioeconomic status
- Sex
- HIV status

1. What do you understand about frailty (being weak)?
2. Do you think those with HIV have better or worse experiences with ageing or frailty?
3. **Beliefs towards ageing and NCDs**
4. What do you feel are some of the gains and losses that come with ageing?

***Probe***

- *Work*
- *Disease*
- *Loneliness*
- *Wisdom*

1. When it comes to your health, what outcomes matter the most to you?
2. How do you feel about being a participant of the study and getting screened for NCDs?
   1. Why do you feel this way?
